# Classification of Human Daily Activities Using Ensemble Methods Based on Smartphone Inertial Sensors

**DOI:** 10.3390/s18124132

**Published:** 2018-11-26

**Authors:** Ku Nurhanim Ku Abd. Rahim, I. Elamvazuthi, Lila Iznita Izhar, Genci Capi

**Affiliations:** 1 Smart Assistive and Rehabilitative Technology (SMART) Research Group, Department of Electrical and Electronic Engineering, Universiti Teknologi PETRONAS, 32610 Bandar Seri Iskandar, Malaysia; ku_g03269@utp.edu.my (K.N.K.A.R.); lila.izhar@utp.edu.my (L.I.I.); 2 Assistive Robotics Laboratory, Department of Mechanical Engineering, Faculty of Science and Engineering, HOSEI University, Tokyo 184-8584, Japan; capi@hosei.ac.jp

**Keywords:** gait, human activity recognition, smartphone, wearable sensor, human daily activity, ensemble method

## Abstract

Increasing interest in analyzing human gait using various wearable sensors, which is known as Human Activity Recognition (HAR), can be found in recent research. Sensors such as accelerometers and gyroscopes are widely used in HAR. Recently, high interest has been shown in the use of wearable sensors in numerous applications such as rehabilitation, computer games, animation, filmmaking, and biomechanics. In this paper, classification of human daily activities using Ensemble Methods based on data acquired from smartphone inertial sensors involving about 30 subjects with six different activities is discussed. The six daily activities are walking, walking upstairs, walking downstairs, sitting, standing and lying. It involved three stages of activity recognition; namely, data signal processing (filtering and segmentation), feature extraction and classification. Five types of ensemble classifiers utilized are Bagging, Adaboost, Rotation forest, Ensembles of nested dichotomies (END) and Random subspace. These ensemble classifiers employed Support vector machine (SVM) and Random forest (RF) as the base learners of the ensemble classifiers. The data classification is evaluated with the holdout and 10-fold cross-validation evaluation methods. The performance of each human daily activity was measured in terms of precision, recall, F-measure, and receiver operating characteristic (ROC) curve. In addition, the performance is also measured based on the comparison of overall accuracy rate of classification between different ensemble classifiers and base learners. It was observed that overall, SVM produced better accuracy rate with 99.22% compared to RF with 97.91% based on a random subspace ensemble classifier.

## 1. Introduction

Recently, increasing interest has been shown in analysing human gait using wearable sensors, which is known as Human Activity Recognition (HAR). HAR with automatic recognition of physical activities is increasingly being studied and applied in Human-Computer Interaction (HCI), mobile and pervasive computing. One of the objectives of HAR is to offer information that enables computing frameworks to productively aid users in their tasks [1]. A few feasible applications that can be used with HAR which improves the service are on-request data systems, monitoring and surveillance system of smart homes, interactive interfaces for mobile services and games, and medical care service applications for both inpatient and outpatient treatments [2,3,4]. In addition, other applications include bilateral links targeted to advertising, entertainment, games, and multimedia visualization guidance [5,6].

Typically, human daily activities are divided into three categories, namely, gestures, low-level activities and high-level activities. Gestures involve simple activities such as the opening-closing of hands and bending of arms. The low-level activities are standing, sitting, walking, cycling and jogging, whereas, the high-level activities are cooking, dancing, eating, drinking and talking. A number of researchers have explored machine vision systems in gesture and activity recognition from video and still images in various settings [7,8,9].

Advances in sensor innovation in on-body wearable sensors and smartphones have enabled them to be used effectively for HAR systems. However, the difference between on-body wearable sensor-based HAR systems and the smartphone-based HAR systems is that, smartphones have a few integrated sensors that are equipped to provide an extensive variety of selections embedded into one cohesive device. Moreover, smartphones have computing ability, although not as capable as the devoted control units of wearable-sensor systems. In addition, smartphones have become an essential gadget in human’s daily life and the usage of a smartphones greatly exceeds that of on-body wearable sensor-based systems. Hence, smartphones have become a prominent tool for assisting and supporting patients undergoing health rehabilitation and treatment, activity monitoring of daily living and diets, and for numerous other health issues [10,11].

One of the critical issues in HAR is the classification of the different activities performed by the users. The studies conducted in past show that machine learning algorithms such as Naïve Bayes tree (NBTREE), Decision Trees (C4.5), Neural Network (NN), k-Nearest Neighbour (k-NN) and Support Vector Machine (SVM) have been used for classification employing smartphone data [12,13,14,15]. Recently, Ensemble learning with bagging and boosting techniques have been found to enhance the accuracy of classifiers. Ensemble learning has been effectively tested and validated adequately on various datasets [16,17].

The study described in this paper highlights the classification of six different daily activities based on the data acquired from inertial sensors of a smartphone using ensemble methods such as Bagging, Adaboost, Rotation forest, Ensemble nested dichotomies (END) and Random subspace together with two base learner techniques such as SVM and Random forest (RF). Two datasets from the UCI Machine Learning Repository [18] were utilised in the study, where each dataset involved 30 subjects performing activities such as walking, walking upstairs, walking downstairs, sitting, standing and laying. The classifications have been evaluated in terms of accuracy, precision, recall, F-measure, and receiver operating characteristic (ROC) curve. This paper is structured as follows: Section 2 discusses the literature review in terms of related work and general HAR system. Methods are presented in Section 3, followed by Section 4 which describes the results and discussion, and finally, conclusions are provided in Section 5.

## 2. Literature Review

### 2.1. Related Work

Over the last few years, from 2013 to 2018, human daily activities such as sitting on chairs and on the floor, lying right, lying left, slow walk, brisk walk, walking upstairs, walking downstairs, standing, laying, etc. have been extensively studied. Many researchers have worked towards evaluation of HAR using the smartphone with different daily activities. These activities are classified based on feature extraction schemes that are broadly categorized as time and frequency domains. In [19,20,21,22,23,24,25] researchers have implemented the time domain and frequency domain feature extraction as a combined approach. Other researchers in [26,27,28,29,30,31] have used feature extraction in the time domain only, whilst, in [32] researchers applied the frequency domain and time-frequency domain. The authors in [33] have chosen the time domain, frequency domain and time-frequency domain for feature extraction. Out of these studies, Saha et al. [19] found that the ensemble classifier performs best, with an overall accuracy rate of 94% using accelerometer and gyroscope sensor data. In the research carried out by Mohamed et al. [20], a combination of accelerometer data from the arm, belt and pocket analysed using rotation forest with the base learner C4.5, was found to provide the best overall classification accuracy rate of 98.9% [20]. Researchers in [21,32], and [23,24,25] have analyzed the same dataset. Ronao and Cho [21] found that classification using two stages of continuous hidden Markov model (TS-CHMM) achieved the highest overall accuracy rate of 93.18%. Jiang, Yin et al. [32] have obtained the best overall accuracy rate of 97.59% using deep convolution neural network (DCNN). Research reported by Kastner et al. [23] provided the best results with an overall classification accuracy rate of 96.23% with generalized learning vector quantization (GLVQ). Romero et al. [24] have found that One vs. one (OVO), OVO-SVM gives the best overall classification accuracy rate as 96.4%; whilst, Anguita et al. [25] managed to gain a little improvement and reported best overall accuracy rate as 96.5% for classification using One vs. all (OVA), OVA-SVM. Researchers in [26,27,28], have analyzed the same dataset for walking, jogging, walking downstairs, walking, upstairs, sitting, and standing activities. A study conducted by Sufyan et al. [26] found that classification on voting Multilayer perceptron (MLP) and NBtree give the best accuracy rate for classification based on each activity. This study found that Voting MLP-NBtree gives the best accuracy rate of classification on walking at 99.23%, jogging at 98.86%, walking upstairs at 93.35%, walking downstairs at 90.15%, sitting at 98.37% and standing at 98.37%. Research by Daghistani and Alshammari [27] found the best overall classification accuracy rate using Adaboost (J48) at 94.034%, whilst, Catal et al. [28] reported the highest overall classification accuracy rate based on voting (J48, logistic regression and MLP) as 94.06%. Gupta and Kumar [29] stated the study on human activities on sitting, standing, walking and running produced the best overall accuracy rate of 98.83% using an Adaboost classifier. Research by Gao et al. [33] showed that C4.5 was the best classifier with a 96.4% overall accuracy rate for lying, sitting, standing, walking and transition activity. Bayat et al. [30] studied HAR comparison between different classifiers such as MLP, SVM, RF, simple logistic, logitboost, Logistic model tree (LMT) and voting classifier with the triaxial accelerometer data in a smartphone that placed in pocket and hand. The results of this study showed that data in hand produces best overall classification accuracy rate using voting combination of MLP, logitboost and SVM classifier with 91.15%, whilst, data in pocket gives best accuracy of classification rate as 90.34% using voting combination of MLP, RF and simple logistic. Research studies from Ha and Ryu [31] have reported that the best overall classification accuracy rate of 97.8% was obtained with an ensemble method known as Error correcting output coding (ECOC) that was combined with the random forest as the base learner. Overall, it can be concluded that Ensemble methods produce better results compared with other algorithms.

### 2.2. HAR System

**Figure 1:**
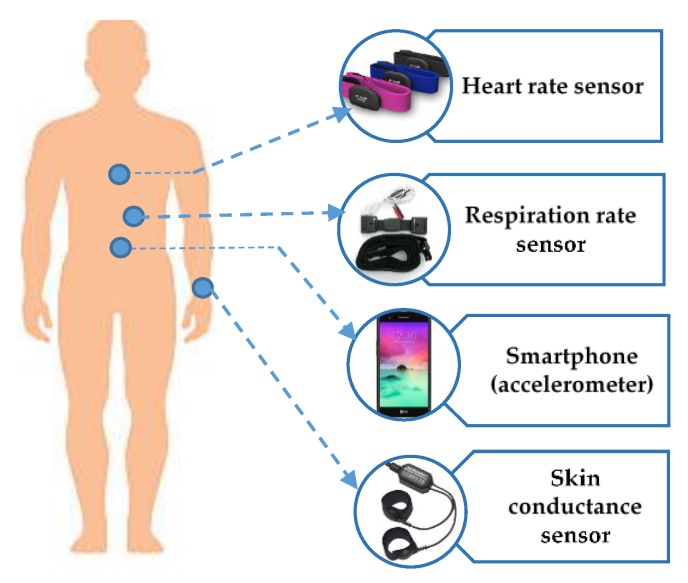
Wearable sensors for human daily activities.

HAR is a way toward recognizing common human activities in daily living. It is turning into an attractive research field because of numerous areas of application. Physiological signals, environmental signals, location data and acceleration signals are the categories of input data that are acquired from wearable sensors in an HAR setting. Physiological signals data such as skin conductivity, heart rate, skin temperature, respiration rate and electrocardiography signals have also been considered in a few research studies to improve the recognition accuracy [34]. Environmental signals, for example, audio level, temperature, humidity are proposed to provide information exposing the individual’s environment [35,36]. The Global Positioning System (GPS) empowers all kinds of area-based information. Current smartphones are equipped with integrated GPS, making it exceptionally advantageous for context-aware applications such as the recognition of the individual’s transportation mode [37]. Accelerometers can be categorized into two types: either as body wearable sensors or incorporated with new models of mobile devices such as smartphones and smart watches which record the body movement [38,39]. The majority of research work in HAR applications is performed using wearable sensors. Figure 1 shows the HAR with different wearable sensors.

**Figure 2:**
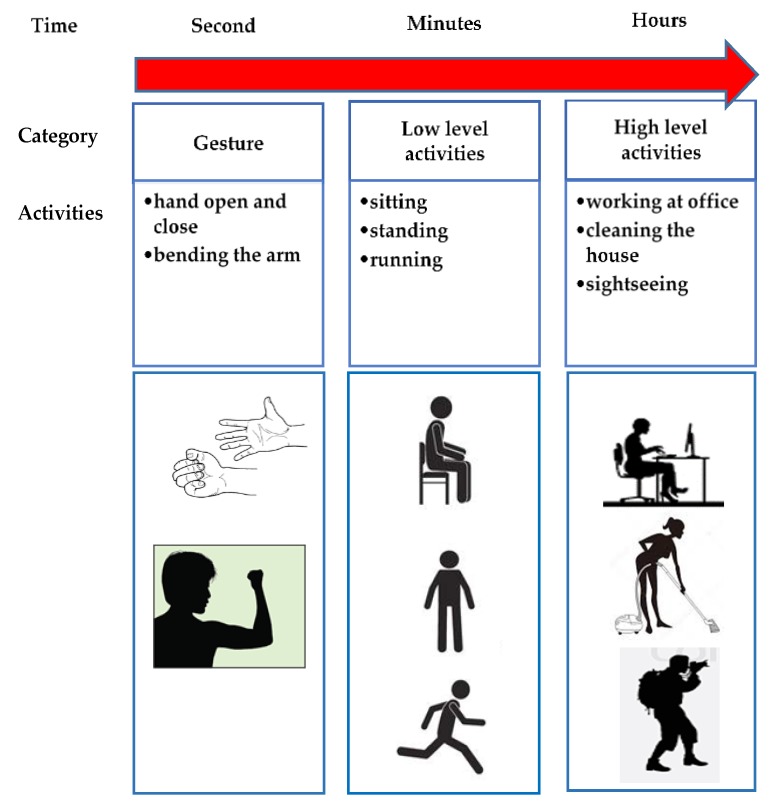
Categories of human daily activities.

The various types of wearable sensors are used to identify different human activities in three categories: gesture, low-level activities and high-level activities as shown in Figure 2.

Gestures activities are classified as extremely short activities such as bending the arm, or opening and closing the hands. Human daily activities such as sitting, standing and running which typically last between seconds and a few minutes are known as low-level activities. Activities like working at the office, cleaning the house and sightseeing normally last for more than a few minutes up to a few hours are known as high-level activities [40]. Detailed information about the general process for training and testing the data of HAR systems based on wearable sensors can be found in [41].

## 3. Methods

### 3.1. Signal Processing Method for HAR

**Figure 3:**
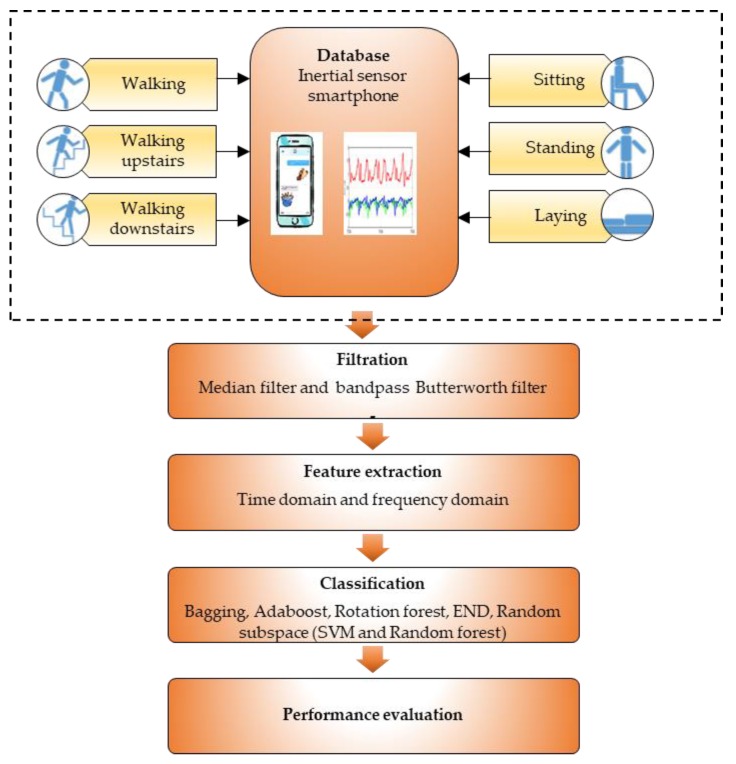
Signal processing method of HAR system.

The signal processing method of HAR system that was carried out in this research is shown in Figure 3.

According to Figure 3, the raw signals of human daily activities such as walking, walking upstairs, walking downstairs, sitting, standing and laying are acquired from the inertial sensors of smartphones. Then, the raw signals are segmented using sliding windows and filtered using median and band pass Butterworth filters to remove the irrelevant information or noise. Next, feature extraction of time and frequency domain are implemented. Thereafter, all the data of extracted features are classified with ensemble classifiers using base learners of SVM and RF. The performance of classification of is measured using performance evaluation metrics such as precision, recall, F-measure, accuracy and ROC.

### 3.2. Database

For this study, two datasets were obtained from UCI Machine Learning Repository [18]. The datasets consist of dataset 1 and dataset 2, containing 5447 data samples and 10,299 data samples, respectively. Dataset 1 was collected from a smartphone worn around the waist of 31 participants within the age group of 22–79 years. Each activity was performed for 60 s. For dataset 2, a group of 30 volunteers with ages ranging 19–48 years were selected. Each person was instructed to follow a protocol of activities while wearing a waist mounted smartphone. For both datasets, the data collections were performed using Samsung Galaxy S2 smartphones which comprise an accelerometer and gyroscope to acquire tri-axial linear acceleration and angular velocity signals at a sampling rate of 50 Hz. Although the tasks were carried out in laboratory conditions, volunteers were asked to perform freely the sequence of activities aiming to simulate a more naturalistic dataset. Table 1 shows the activities that were carried out for both the datasets.

### 3.3. Data Pre-Processing Filtering and Feature Extraction

The raw signals were processed by applying a median filter and 3rd order low pass Butterworth filter with 20 Hz cut-off. These signals were then segmented with fixed width sliding windows of 2.56 s with 50% overlap. A total of 17 features extracted from the time and frequency domains for each window were used in this work, where only about six features are shown in Table 2. The full 17 features can be found in [42].

### 3.4. Classification Techniques—Ensemble Methods

The principle thought behind the ensemble method is to evaluate a few single classifiers and combine them to acquire a classifier that surpasses each individual one of them. The motivation behind supervised learning is to classify patterns or instances that are specified into a group denoted as labels or classes. Often, the classification is determined by a classification models (classifiers) that are inferred from a pre-classified design model. Nevertheless, the classification employs knowledge that is provided by a specialist in the application area which is referred to as training data. The training set is a standardized supervised learning set that has a set of instances. The labels of the instances in the training set are identified and the objective is to develop a model with a specific end goal to label new instances. An algorithm which builds the model is called inducer and a case of an inducer for a specific training set is known as a classifier [43]. An ensemble comprises several inducers which are commonly referred to as the base classifier or base learner. A base learner is an algorithm that receives a set of labelled instances as input and produces a model that simplifies these instances. Predictions are determined for new unclassified instances by utilizing the created model. The generalization capability of an ensemble is usually more robust than that of base learners. As a matter of fact, ensemble method is likable because it can boost weak learners, referred to base classifiers which are marginally better than random estimate to strong learners which can make very accurate predictions. In this research work, ensemble methods such as Bagging, Adaboost M1, Rotation forest, Ensembles of Nested Dichotomies (END) and random subspace were used for classification. An ensemble inducer can be of any type of base classifiers such as decision tree, neural network, k-NN and others type of base learner algorithm [43]. In this research work, the base learners applied were SVM and RF. The detail information about the ensemble methods can be found in [44] for bagging, [45,46] for Adaboost.M1, [47] for Rotation Forest, [48] for Ensembles Nested Dichotomies (END), [49] for Random subspace, [50] for Random Forest (RF) and [51] for SVM.

The above stated five Ensemble methods and two base learner algorithms were used to classify six human daily activities based on a classifier tool known as WEKA 3.8 Version [52] with model evaluation of the holdout method (contains 70% of the training set and 30% of the test set) and 10-fold cross-validation method. A Wilcoxon test was performed on the results to discover if the accuracy rate of classification instances was significantly different for SVM as a baseline compared to the RF as base learners in five different ensemble methods. This statistical test was conducted on IBM SPSS version 20 [53]. A value of *p* less than 0.05 is considered as statistically significant when the confidence level is set to 95%.

### 3.5. Performance Evaluation

The performance evaluation that was implemented in this study is based on the following expressions:

(1) $$\mathrm{Precison}=\frac{\mathrm{TP}}{\mathrm{TP}+\mathrm{FP}}$$

(2) $$\mathrm{Recall}=\frac{\mathrm{TP}}{\mathrm{TP}+\mathrm{FN}}$$

(3) $$F-\mathrm{measure}=\frac{2\;\left(\mathrm{TP}\right)}{2\;\left(\left(\mathrm{TP}\right)+\left(\mathrm{FN}\right)\right)}$$

(4) $$\mathrm{Accuracy}=\frac{\mathrm{TP}+\mathrm{TN}}{\mathrm{TP}+\mathrm{TN}+\mathrm{FP}+\mathrm{FN}}$$

Receiver operating characteristic (ROC) curve is a plot graph of the true positive rate against the false positive rate at different classification threshold setting. The true positive rate is also known as Recall in Equation (2) and false positive is defined as following expression:

(5) $$\mathrm{False}\;\mathrm{positive}\;\mathrm{rate}=\frac{\mathrm{FP}}{\mathrm{FP}+\mathrm{TN}}$$

where TP—True Positive, TN—True Negative, FP—False Positive, and FN—False Negative.

## 4. Results and Discussion

### 4.1. Performance Evaluation of Dataset 1

#### 4.1.1. Holdout and 10 Cross-Validations for Precision, Recall, F-measure, and ROC Evaluation

Table 3 presents the performance evaluation of END classifier with SVM and RF as base learner including precision, recall, F-measure and receiver operating characteristic (ROC) as the best classifier of the holdout method.

As shown in Table 3, the results of END classifier evaluation on holdout method obtained the best precision in activity A6 with 100% using SVM as a base learner compared to RF at 99.1%. For activity A5, both SVM and RF base learners achieved the precision results at 88.8%. But in activity A4, better precision was obtained by RF with 96.6% followed by SVM with 92.8%. For A3, A2 and A1, precision results show that SVM has given higher result range of 95.3% to 96.1% compared to RF with 89.6% to 92.7%. Recall results show that SVM obtained 99.10% and RF obtained 97.30% for activity A6. However, for activity A5, RF produced 94.6% compared to SVM with 91.5%. For A4, A3, A2, and A1, recall results show that SVM produced results ranging from 90.4% to 97.6% compared to RF with 90.1% to 94.9%. Results of F-measure evaluation for activity A6 is 99.6% for SVM and 98.2% for RF. However, the F-measure results for activity A4 and A5 for RF are 93.2% and 91.6% respectively compared to SVM with 91.60% and 90.20%. Activities A1, A2, and A3 give SVM better F-measure results ranging from 94.7% to 96.9%, higher than RF range from 90.2% to 93.8%. RF gained greater results for ROC evaluation with results ranging from 99.4% to 100% compared to SVM which produced 95.2% to 99.8% for all activities. Table 4 presents the performance evaluation of Random subspace classifier with SVM and RF as base learner for 10-fold cross-validation method.

As shown in Table 4, the results of Random subspace classifier using 10-fold cross-validation indicates SVM as base learner produces better precision range of results from 95.6% to 99% compared to RF with 90.3% to 98% for activities A1, A2, A3 and A6. However, RF has obtained better precision results of 94.2% and 95.9% compared to SVM with 93% and 93.5% for activities A4 and A5. For recall, activities A1, A2, A3, and A6 with SVM produced superior results from 94.7% to 99.4% compared to RF with 90% to 98.2%. However, RF has obtained better recall results of 94.2% and 95.9% compared to SVM with 93.9% and 92.5% for activities A4 and A5. The results of F-measure for activities A1, A2, A3 and A6 ranged from 95.1% to 99.2% for SVM and 91.2% to 98.1% for RF. The ROC results for RF were from 99.3% to 100% and 98.2% to 99.8% for SVM for all the activities. In dataset 1, overall results of 10-fold cross-validation model evaluation give better results compared to holdout for both base learners.

#### 4.1.2. Holdout and 10-Fold Cross-Validations for Overall Accuracy Rate

**Figure 4:**
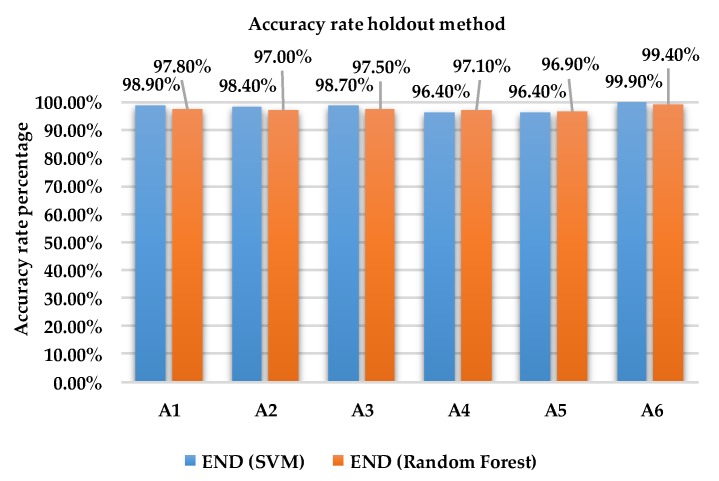
Accuracy rate of each activity holdout method dataset 1.

**Figure 5:**
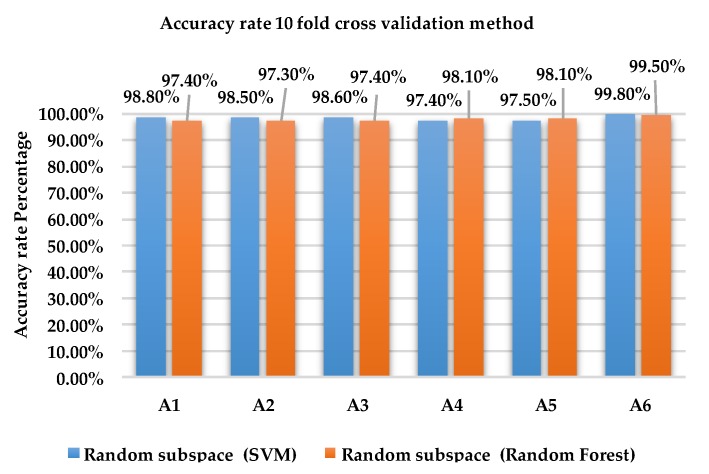
Accuracy rate of each activity 10 fold cross validation method dataset 1.

Figure 4 and Figure 5 show the accuracy rate classification for each activity for ensemble method with base learners SVM and RF using holdout and 10-fold cross validation methods, respectively, for dataset 1.

As shown in Figure 4, the accuracy rate of activity A1, A2, A3 and A6 of Random subspace classifier with SVM for holdout method produced superior accuracy rate with the range from 98.4% to 99.9% compared to RF which produced an accuracy rate of 97.0% to 99.4%. On the other hand, RF produced better results with an accuracy rate of 97.1% and 96.9% compared to SVM with 97.1% and 96.9% activities A4 and A5.

Results from Figure 5 indicate that the accuracy rate for activities A1, A2, A3 and A6 of Random subspace classifier with SVM using 10-fold cross validation is higher with 98.5% to 99.8% compared to RF with 97.3% to 99.5%. The accuracy rate for activities A4 and A5 is 97.4% 97.5% for SVM and 98.1% for RF.

Table 5 and Table 6 show the overall accuracy rate of all other ensemble methods with base learners SVM and RF using holdout method and 10-fold cross validation method for dataset 1.

Results in Table 5 demonstrate that SVM has a significantly greater accuracy with Bagging (93.83%, *p* = 0.028). There were no significant differences for other ensemble methods using the holdout method and 10-fold cross-validation method as shown in Table 6 for dataset 1.

### 4.2. Performance Evaluation of Dataset 2

#### 4.2.1. Holdout Method for Precision, Recall, F-measure, and ROC Evaluation

Table 7 presents the performance evaluation of random subspace classifier with SVM and RF as base leaners as the best classifier of holdout method.

As shown in Table 7, the results of random subspace classifier evaluation on holdout method obtained the best precision in activity A6 with 100% for SVM and RF. For other activities, SVM shows better precision results between 96.7% and 99.8% compared to RF which obtained 95.8% to 98.8%. For recall, SVM achieved 100% compared to RF 99.8% for activity A6. SVM results for activities A1, A2, A3 and A4 ranges from 97% to 100% compared RF which obtained 95.8% to 99%. However, RF produced better recall results with 97.2% compared to SVM which recorded 97% for activity A5. The F-measure evaluation shows that SVM obtained 100% compared to RF that produced 99.9% for activity A6. For activity A4, SVM and RF obtained 97% and 95.9% respectively which are the lowest performance compared to activities A1, A2, A3 and A5. Table 8 shows the ROC curve of each activity of Random subspace with SVM classifier and Random subspace with RF using hold out method for dataset 2.

As shown in Table 8, the results of ROC evaluation gained 1.000 in activities A1, A2, A3 and A6 in both base learners. For activities A4 and A5, the ROC results for RF was 0.999 compared to 0.995 and 0.998 for SVM.

#### 4.2.2. 10-Fold Cross Validation for Precision, Recall, F-measure, and ROC Evaluation

The cross-validation method evaluates fold (k) = 10 for all activities of dataset 2. Table 9 presents the performance evaluation of Random subspace as the best classifier of 10-fold cross-validation method.

From Table 9, the precision results for random subspace classifier on 10-fold cross-validation method shows that SVM and RF obtained 99.9% for activity A1 and 100% for both the SVM and RF for activity A6. For activities A2, A3, A4, and A5, SVM obtained between 97.9% and 99.9% and RF 95.6% to 98.5%. Recall results achieved 100% for activities A1 and A6 using SVM compared to RF which received 98.4% and 99.8%. For activities A2, A3, A4, A5, SVM gained between 98% and 99.8%, whereas, RF obtained between 95.2% and 99.2%. The results of F-measure for activities A1 and A6 for SVM was 100% compared to RF which obtained between 98.7% and 99.9%. SVM obtained between 98% and 99.7% compared to RF which produced results between 96.1% and 98.3% for the results of F-measure in activities A2, A3, A4, and A5.

Table 10 shows the ROC of each activity of Random subspace with SVM and Random subspace with RF classifier using 10-fold cross validation method dataset 2.

As shown in Table 10, the results of ROC evaluation for activities A1, A2, A3, and A6 for SVM is 1.000, whereas, RF produced 1.000 only for A6. For activities A4 and A5, SVM recorded 0.999. RF achieved 0.998 for activity A4 and 0.999 for A1, A2, A3 and A5.

As can be seen in Table 7, Table 8, Table 9 and Table 10, overall performance evaluation in each activity on Random subspace classifier with base learner SVM is better than RF for holdout method and 10-fold cross-validation methods. The 10-fold cross-validation model evaluation gives better results compared to holdout for both base learners.

**Figure 6:**
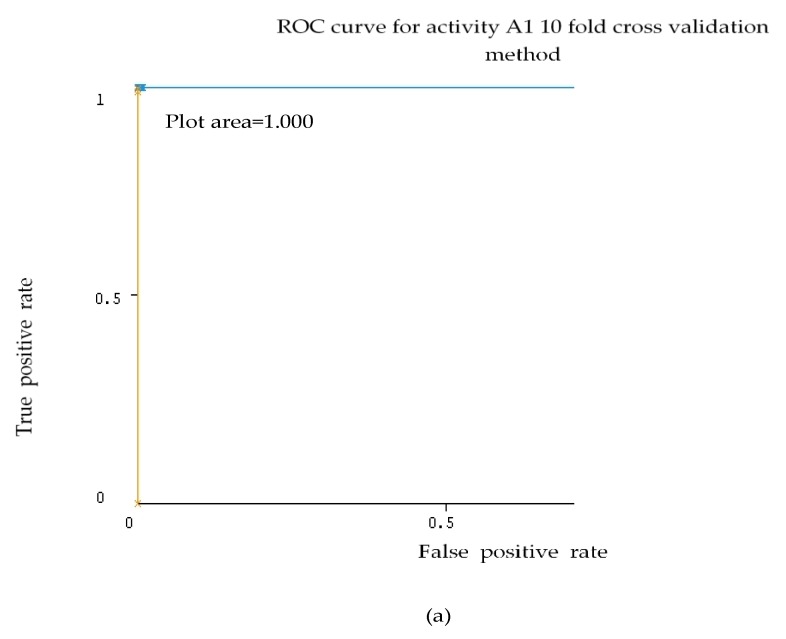

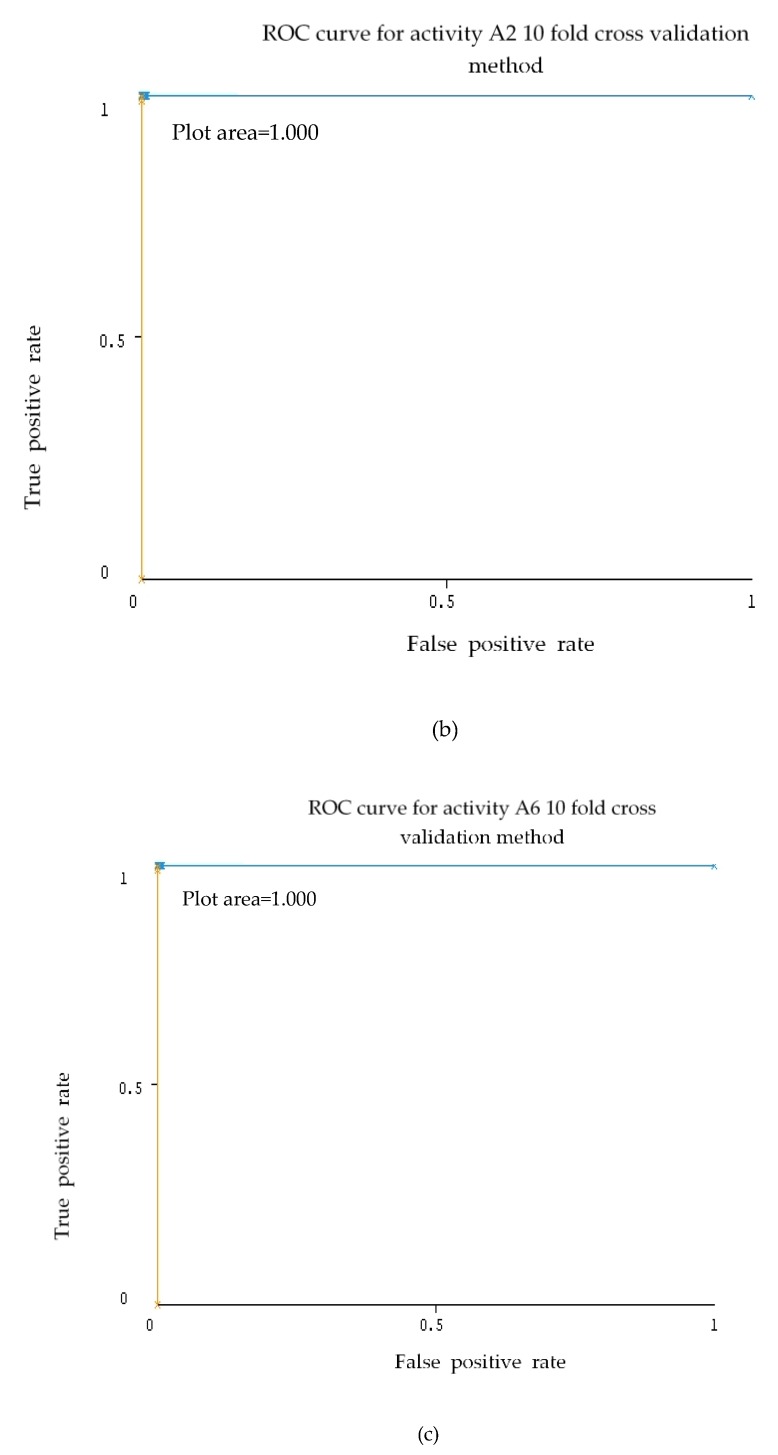
ROC graph activity A1 (**a**), A2 (**b**) and A6 (**c**) of Random subspace with SVM classifier using 10 fold cross validation method dataset 2.

**Figure 7:**
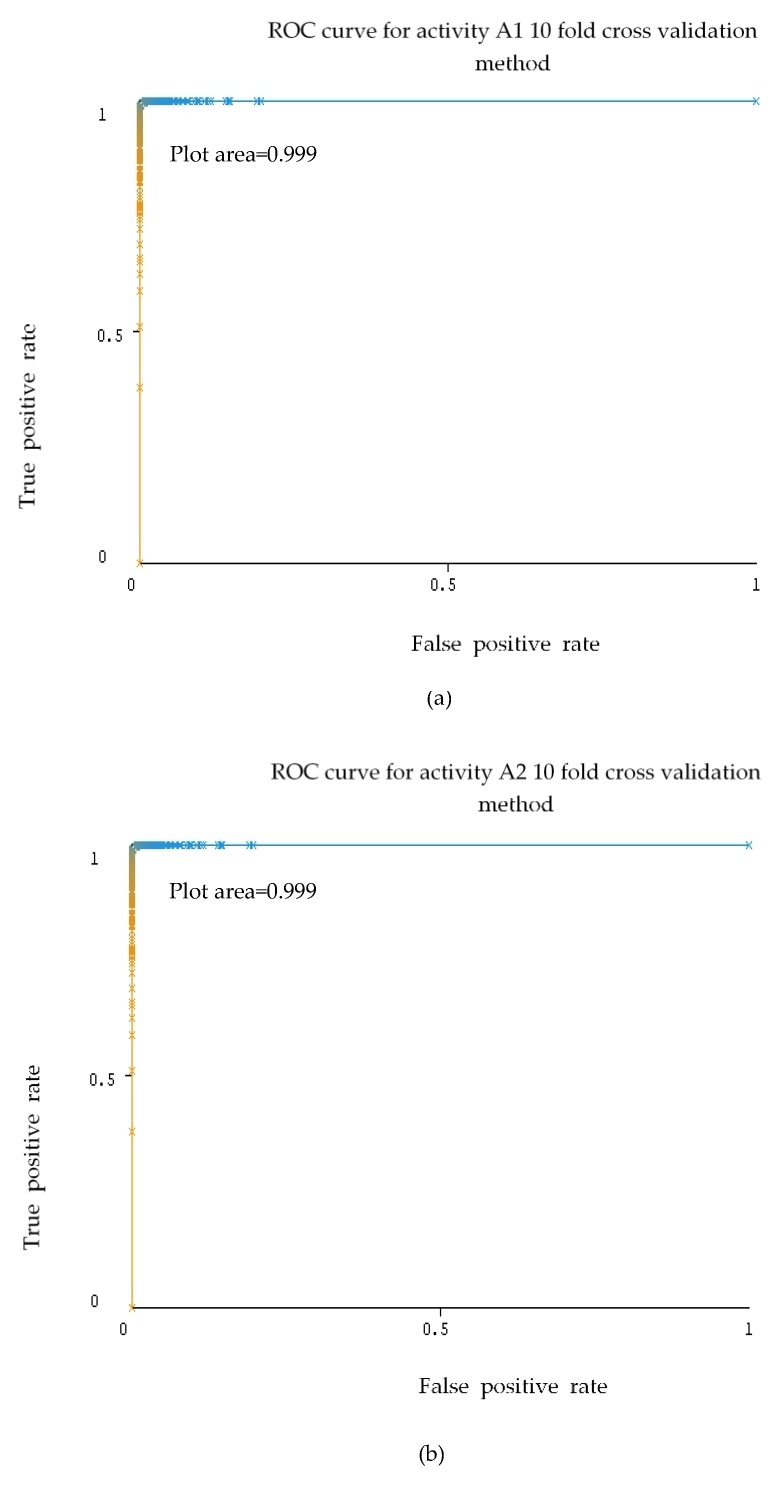

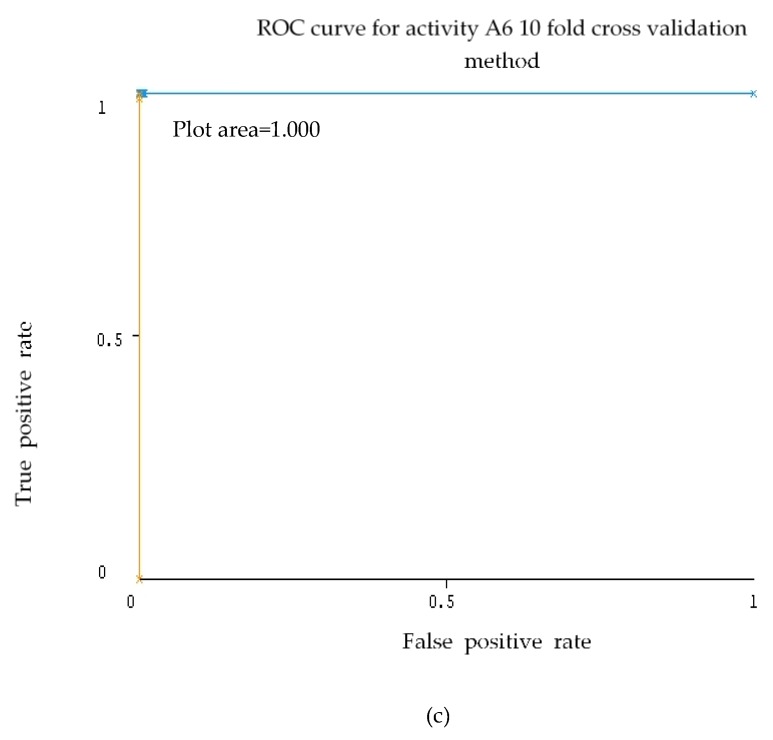
ROC graph activity A1 (**a**), A2 (**b**) and A6 (**c**) of Random subspace with RF classifier using 10-fold cross validation method dataset 2.

Representative ROC curves for SVM and RF are shown in Figure 6 and Figure 7 based on Table 10 for activities A1, A2 and A6.

As can be seen in Figure 6 and Figure 7, the activities A1, A2 and A6 for SVM and RF, the ROC results produced 1.000. For activities A1 and A2, the ROC results are 0.999 for RF.

#### 4.2.3. Holdout and 10-Fold CROSS-validations for Overall Accuracy Rate Classification

**Figure 8:**
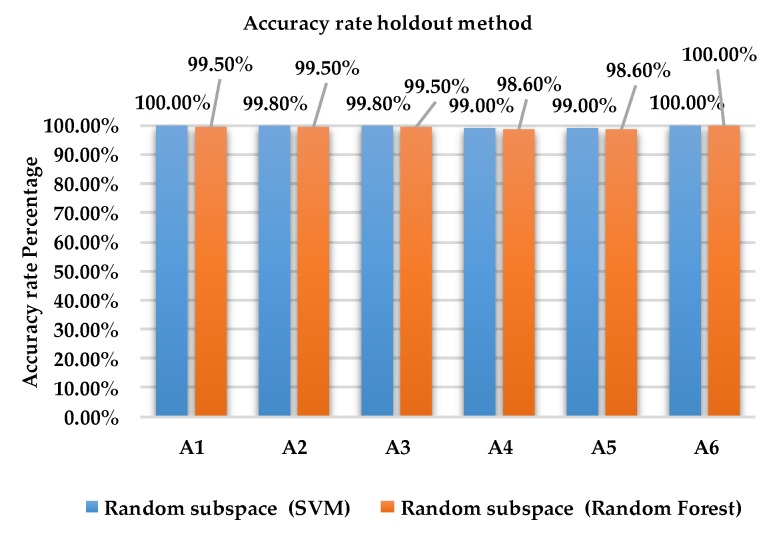
Accuracy rate of each activity for holdout method using dataset 2.

**Figure 9:**
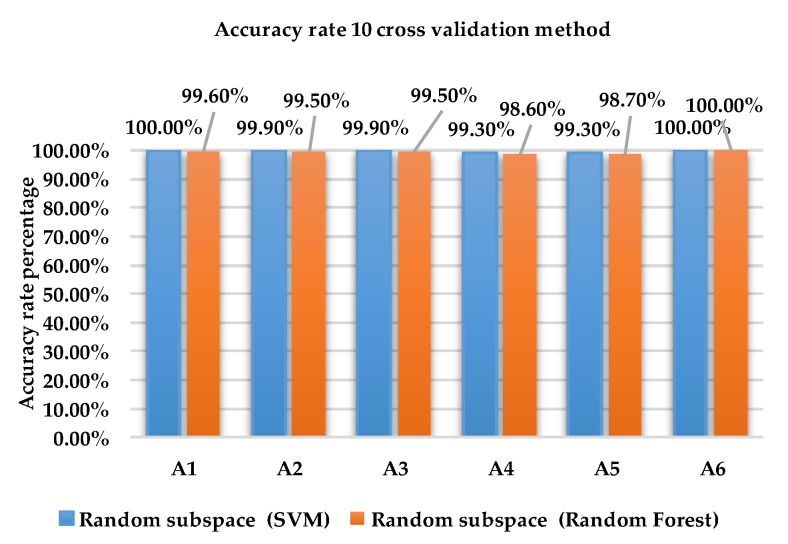
Accuracy rate of each activity of 10-fold cross validation method using dataset 2.

Figure 8 and Figure 9 show the accuracy rate of classification for each activity for ensemble method with base learners SVM and RF using holdout method and 10-fold cross validation method for dataset 2.

As shown in Figure 8, the accuracy rate of activity A6 is 100% for Random subspace classifier with SVM and RF using the holdout method. For activities A1, A2, A3, A4 and A5, the accuracy rate for SVM is between 99% and 100% compared to RF which obtained results between 98.6% and 99.5%.

As shown in Figure 9, the accuracy rate of activity A6 is 100% for Random subspace classifier with SVM and RF using the 10-fold cross validation method. For activities A1, A2, A3, A4 and A5, the accuracy rate for SVM is between 99.9% and 100% compared to RF which obtained results between 98.7% and 99.6%.

Table 11 and Table 12 present the overall accuracy rate classification of other ensemble methods with base learners SVM and RF on holdout method and 10-fold cross validation method for dataset 2.

Results in Table 11 show that SVM base learner has significantly greater accuracy rate than RF with the same probability value (*p*-value) when using Bagging (98.54%, *p* = 0.028), END (98.61%, *p* = 0.028) and Random subspace (98.74%, *p* = 0.028). In this case, random subspace gives the highest accuracy rate in the holdout method. There was no significant difference in accuracy between the SVM and RF when employing Adaboost and RF for holdout method because of the higher *p*-value. From Table 12, SVM base learner demonstrates significantly greater accuracy rate than RF, whilst the random subspace gives the highest accuracy rate with (99.22%, *p* = 0.028). The results of END (99.20%, *p* = 0.028), Adaboost (99.17%, *p* = 0.028) and Bagging (99.07%, *p* = 0.028) have significantly greater overall accuracy rate than RF as a base learner in 10-fold cross-validation method.

### 4.3. Comparative Analysis

The comparison of the overall accuracy rate of classification between different methods of classification with previous research work is represented in Table 13.

As shown in Table 13, the proposed classifier with Random subspace-SVM achieved an accuracy rate of 99.22% for 10-fold cross-validation and 98.74% for holdout method. These proposed classifiers show improvement of overall accuracy rate of classification on the same dataset (10,000 samples) compared to previous work done by Ranoa and Chao [21] that has achieved an overall accuracy rate of 93.18%, Anguita et al. [25] that obtained overall accuracy rate of 96.5%, Romero-Paredes et al. [24] that acquired an overall accuracy rate of 96.4% and Kastner et al. [23] that produced overall accuracy rate of 96.23%. This shows that the Random subspace-SVM ensemble method has the capability to produce higher accuracy due to its ability to find a hyper plane which separates positive and negative training observations, and maximizes the margin between these observations through this hyper plane compared to other methods such as Two stages of continuous Hidden Markov model, OVA MC-SVM-Gaussian kernel, OVO MC-SVM-Linear Kernel majority voting and Kernel generalized learning vector quantization.

Although RF classifiers have predictive performance comparable to that of the best performing alternatives such as SVMs for classification of HAR, nevertheless, this research shows that SVM has a slight edge over RF. From a small sample size (dataset 1) to a larger sample size (dataset 2), the accuracy significantly increased with RF. This is true for SVM as well. This shows that sample size has more impact on the classification accuracy of both the RF and SVM. This is consistent with the results reported by Shao and Lunetta [54], Thanh and Kappas [55], Hasan et al. [56], Solé et al. [57] and Sheshasaayee and Thailambal [58].

It can be established that for the proposed Random subspace-SVM ensemble classification method, the 10-fold Cross-validation evaluation method produced better results than the holdout evaluation method. This is supported by various researchers such as Bengio et al. [59], Kim [60] and Sakr et al. [61].

It can be deduced that the ensemble method gives instinctive, straightforward, well-designed, and strong resolutions for an assortment of machine learning issues. As pointed out by Polikar [16], this method was initially created to enhance classification accuracy by decreasing the modification in classifier outputs. Ensemble methods have since ended up being exceptionally powerful in various areas that are difficult to address utilizing a single model-based classifier. Generally, most ensemble methods are self-determined for the type of base classifier used to construct the ensemble. This is a significant advantage that permits developing a specific kind of classifier that might be known to be most appropriate for a certain application.

## 5. Conclusions

In this study, different ensemble classifiers with different base learner algorithms were implemented to classify six human daily activities based on tri-axial inertial smartphone data. Comparative studies of classification techniques were presented using Bagging, Adaboost, Rotation forest, END and Random subspace with base learner as SVM and RF. The performance measures used to evaluate the classification techniques include overall accuracy, precision, recall, F-measure and ROC. Holdout and 10-fold cross-validation evaluation methods were used in the model evaluation of classification. As seen from the obtained results, Random subspace classifier with SVM gives the best results overall accuracy rate over different ensemble classifiers. The comparison in each activity classification showed the overall results performance of precision, recall, F-measure and ROC and accuracy rate using 10-fold cross-validation method was slightly higher compared to the holdout method. It can be summarized that ensemble classifiers have produced improved performance for the HAR with six different activities such as walking, walking upstairs, walking downstairs, sitting, standing and lying. In future, other methods would be explored to improve the performance.

## Figures and Tables

**Table 1:** List of daily living activities.

Activity Reference	Description of Activity
A1	Walking
A2	Walking upstairs
A3	Walking downstairs
A4	Sitting
A5	Standing
A6	Laying

**Table 2:** 6 Features extraction of time and frequency domain for each window.

Feature	Description
Min	Smallest value in the array
Max	Largest value in the array
Std	Standard deviation
Entropy	Signal entropy
Kurtosis	Kurtosis of the frequency domain signal
Skewness	Skewness of the frequency domain signal

**Table 3:** Performance evaluation of each activity with random subspace classifier on the holdout method.

END (Holdout)								
SVM					RF			
Activity	Precision	Recall	F-measure	ROC	Precision	Recall	F-measure	ROC
A1	96.10%	97.60%	96.90%	99.20%	92.70%	94.90%	93.80%	99.70%
A2	95.30%	94.00%	94.70%	98.80%	89.60%	90.80%	90.20%	99.40%
A3	95.50%	96.00%	95.70%	98.80%	92.30%	91.90%	92.10%	99.50%
A4	92.80%	90.40%	91.60%	95.20%	96.60%	90.10%	93.20%	99.60%
A5	88.80%	91.50%	90.20%	96.20%	88.80%	94.60%	91.60%	99.40%
A6	100.00%	99.10%	99.60%	99.80%	99.10%	97.30%	98.20%	100.00%

**Table 4:** Performance evaluation for each activity of a random subspace classifier on 10-fold cross-validation method.

Random Subspace (10-fold Cross-Validation)								
SVM					Random Forest			
Activity	Precision	Recall	F-measure	ROC	Precision	Recall	F-measure	ROC
A1	95.60%	97.70%	96.70%	99.30%	90.30%	95.90%	93.10%	99.60%
A2	95.40%	94.80%	95.10%	98.70%	92.50%	90.00%	91.20%	99.30%
A3	96.60%	94.70%	95.70%	98.30%	93.80%	90.10%	91.90%	99.40%
A4	93.00%	93.90%	93.40%	98.20%	96.40%	94.20%	95.30%	99.80%
A5	93.50%	92.50%	93.00%	98.60%	94.10%	95.90%	95.00%	99.60%
A6	99.00%	99.40%	99.20%	99.80%	98.00%	98.20%	98.10%	100.00%

**Table 5:** Overall performance evaluation ensemble methods on the holdout method.

Overall Accuracy Rate			
Holdout			
Ensemble Method	SVM	RF	*p*-Value
Bagging	93.83%	91.62%	0.028
Adaboost	94.24%	94.24%	0.917
Rotation forest	89.95%	92.23%	0.344
END	94.50%	93.16%	0.172
Random subspace	94.24%	92.76%	0.116

**Table 6:** Overall performance evaluation ensemble classifiers for 10-fold cross-validation method.

Overall Accuracy Rate			
10-Fold Cross-Validation			
Ensemble Method	SVM	Random Forest	*p*-Value
Bagging	94.57%	92.88%	0.173
Adaboost	94.84%	94.74%	0.917
Rotation forest	90.65%	93.65%	0.075
END	95.14%	94.48%	0.249
Random subspace	95.33%	94.08%	0.249

**Table 7:** Performance evaluation of each activity with random subspace classifier on the holdout method.

Random Subspace (Holdout)						
SVM				RF		
Activity	Precision	Recall	F-measure	Precision	Recall	F-measure
A1	99.80%	100.00%	99.90%	98.20%	99.00%	98.60%
A2	98.90%	99.50%	99.20%	98.20%	98.60%	98.40%
A3	99.80%	99.00%	99.40%	98.80%	97.30%	98.00%
A4	96.70%	97.20%	97.00%	96.60%	95.30%	95.90%
A5	97.70%	97.00%	97.30%	95.80%	97.20%	96.50%
A6	100.00%	100.00%	100.00%	100.00%	99.80%	99.90%

**Table 8:** ROC for each activity of the random subspace classifier on holdout method.

Random Subspace (Holdout)		
	SVM	RF
Activity	ROC	ROC
A1	1.000	1.000
A2	1.000	1.000
A3	1.000	1.000
A4	0.995	0.999
A5	0.998	0.999
A6	1.000	1.000

**Table 9:** Performance evaluation for each activity of the random subspace classifier on 10-fold cross-validation method. Random subspace (10-fold cross-validation method).

Random Subspace (10-Fold Cross-Validation Method)						
SVM				RF		
Activity	Precision	Recall	F-measure	Precision	Recall	F-measure
A1	99.90%	100.00%	100.00%	99.90%	98.40%	98.70%
A2	99.70%	99.70%	99.70%	97.50%	99.20%	98.30%
A3	99.70%	99.80%	99.80%	98.50%	97.60%	98.00%
A4	97.90%	98.00%	98.00%	97.00%	95.20%	96.10%
A5	98.20%	98.10%	98.10%	95.60%	97.30%	96.40%
A6	100.00%	100.00%	100.00%	100.00%	99.80%	99.90%

**Table 10:** ROC for each activity of the random subspace classifier on 10-fold cross validation method.

Random Subspace (10-Fold Cross Validation)		
	SVM	RF
Activity	ROC	ROC
A1	1.000	0.999
A2	1.000	0.999
A3	1.000	0.999
A4	0.999	0.998
A5	0.999	0.999
A6	1.000	1.000

**Table 11:** Overall performance evaluation of ensemble classifiers for the holdout method.

Overall Accuracy Rate			
Holdout			
Ensemble Method	SVM	RF	*p*-Value
Bagging	98.54%	97.18%	0.028
Adaboost	98.43%	98.07%	0.686
Rotation forest	98.07%	98.03%	0.893
END	98.61%	98.03%	0.028
Random subspace	98.74%	97.86%	0.028

**Table 12:** Overall performance evaluation of ensemble methods for the 10-fold cross-validation.

Overall Accuracy Rate			
10-Fold Cross-Validation			
Ensemble Method	SVM	RF	*p*-Value
Bagging	99.07%	97.43%	0.028
Adaboost	99.17%	98.82%	0.028
Rotation forest	98.43%	98.22%	0.249
END	99.20%	98.28%	0.028
Random subspace	99.22%	97.91%	0.028

**Table 13:** Comparison of overall accuracy of classification with previous research work.

Reference	Evaluation Method	Dataset	Classification Method	Overall Accuracy Rate
Proposed classifier	10-fold Cross-validation	10,000 samples	Random subspace-SVM	99.22%
Proposed classifier	Holdout	10,000 samples	Random subspace-SVM	98.74%
Ronao and Cho (2017) [21]	10-fold Cross-validation	10,000 samples	Two stages of continuous Hidden Markov model	93.18%
Anguita et al. (2013) [25]	Holdout	10,000 samples	OVA MC-SVM-Gaussian kernel	96.5%
Romero-Paredes et al. (2013) [24]	Holdout	10,000 samples	OVO MC-SVM-Linear Kernel majority voting	96.40%
Kastner et al. (2013) [23]	Holdout	10,000 samples	Kernel generalized learning vector quantization	96.23%
